# Mechanical properties of muscles and tendon structures in middle-aged and young men

**DOI:** 10.1038/s41598-022-05795-7

**Published:** 2022-02-01

**Authors:** Keitaro Kubo, Toshihiro Ikebukuro, Hideaki Yata

**Affiliations:** 1 Department of Life Science, The University of Tokyo, Meguro, Tokyo, Japan; 2 Sports Science Laboratory, Wako University, Machida, Tokyo, Japan

**Keywords:** Ageing, Musculoskeletal system

## Abstract

The purpose of this study was to compare the mechanical properties of muscles and tendon structures for plantar flexor muscles at various strain rates and jump performances using single joint between middle-aged and young men in order to clarify the mechanisms of age-related decline in power output during vertical jump of middle-aged people previously reported. Passive muscle stiffness of the medial gastrocnemius muscle was determined based on passive muscle force and fascicle length during passive stretching at four angular velocities. Active muscle stiffness was calculated based on changes in muscle force and fascicle length during stretching at five angular velocities after submaximal isometric contractions. Maximal elongation and hysteresis of tendon structures were assessed from estimated muscle force—tendon elongation during ramp and ballistic contractions. Two kinds of unilateral jump heights using only ankle joint (no-countermovement and countermovement jumps) were measured. No significant differences in passive and active muscle stiffness, tendon structure properties (except for maximal elongation during ramp contraction), or jump heights were found between middle-aged and young men. The results suggest that the mechanical properties of muscles and tendon structures for plantar flexor muscles and jump performances using only ankle joint do not show age-related changes in middle-aged men.

## Introduction

It is known that age-associated decline of power is greater than that of muscle strength^1,2^, and the decline in power with aging has already begun by middle age, i.e., around forties^3–6^. Tsubaki et al.^6^ demonstrated in a cross-sectional study that the difference in power during a vertical jump between those in their 20 s and 40 s was greater than that in knee extension isometric strength. According to the findings of a longitudinal study^5^, the rate of decline in power with aging was also greater than that of isometric strength. In addition, Konig et al.^3^ reported that matching middle-aged and young groups for muscle strength eliminated age-related difference in muscular power production during drop jump. As the reasons for these results, age-related changes in the neuromuscular system and atrophy of fast-twitch fibers were proposed to contribute to the decreased power output with aging^3–5^. To the best of our knowledge, however, no reported studies support this speculation, which is tested experimentally in humans.

On the other hand, previous studies using ultrasonography demonstrated that muscular power output was related to the mechanical properties of muscles and tendons as well as neuromuscular factors^7^. Over the last two decades, some studies showed age-related changes in the mechanical properties of human tendons in vivo^8–12^. We previously reported that maximal strain of the Achilles tendon in a group in their 30 s was already lower than that of those in their 20 s, whereas no difference in muscle strength was found between the two groups^11^. To date, few studies have shown age-related changes in tendon hysteresis^8,10^, although tendon hysteresis as well as tendon stiffness affect performance during stretch–shortening cycle exercises^13,14^. In these studies, however, the mechanical properties of tendons were investigated during ramp isometric contraction with a low strain rate (2–7 mm s^−1^^13,15^), and the strain rate of tendons during measurements was markedly lower than that during running and jumping (60–200 mm s^−1^^16,17^). Recent studies showed that tendon properties measured during ballistic contraction (similar to the strain rate of tendons during jumping and sprinting) were markedly different from those during ramp contraction^8,18^. Therefore, we need to investigate age-related changes in tendon properties during ballistic contraction when we are exploring the reasons for a decline in power output of middle-aged people. If a decline in power output of middle-aged people is associated with age-related changes in tendon properties, tendon elongation and hysteresis during ballistic contraction in the middle-aged may already be different from those of young people.

Until now, some studies have investigated the mechanical properties of human muscles based on passive torque and the joint angle during slow passive stretching^19^. According to previous findings^20–23^, passive torque and stiffness of muscles were significantly higher in elderly than young people. Previous studies using animals demonstrated that the amount of collagen of the perimysium and endomysium increased with aging^24^ and age-related increases in cross-linking of collagen fibers were associated with increased passive force and stiffness of aged muscles^25^. To date, passive muscle properties of middle-aged people have remained unknown. In addition, length changes in muscle fibers need to be directly determined to assess passive muscle properties, because changes in the joint angle do not necessarily correspond to those in the muscle fiber length.

However, it is necessary to determine the mechanical properties of muscles under active condition in order to consider the effect of muscle properties on the age-related change in power production during jump. We recently demonstrated that muscle stiffness under active conditions (i.e., active muscle stiffness) could be evaluated according to changes in joint torque and fascicle length during fast stretching^26^. Using this technique, we reported the training-induced changes in active muscle stiffness from cross-sectional and longitudinal studies^18,27,28^. More recently, we were able to determine active muscle stiffness at a high angular velocity (up to 600 deg s^−1^), which corresponded to that during sprinting and jumping^29^. Furthermore, active muscle stiffness at high angular velocities was significantly greater in sprinters than untrained men, whereas there were no differences in active muscle stiffness at low angular velocities between the two groups^30^. Therefore, we expected that active muscle stiffness at a high angular velocity was lower for middle-aged compared with young people, if age-related decline in power in middle-aged people is associated with changes in the mechanical properties of muscles.

The vertical jump tests used in the previous studies as cited earlier^3–6^ consisted of multi-joint movements, so is difficult to specify the muscles acting during tests. Therefore, we need to adopt the test to minimize the contribution of other joints and muscles in order to precisely evaluate the role of muscle and tendon properties. In the present study, we aimed to compare the mechanical properties of muscles and tendon structures (outer-tendon and deep aponeurosis) at various strain rates and jump performances using single joint between middle-aged and young men in order to clarify the mechanisms of age-related decline in power output during vertical jump (i.e., muti-joint exercise) of middle-aged people. We hypothesized that elongation of tendon structures and active muscle stiffness were lower and hysteresis of tendon structures and passive muscle stiffness were higher in middle-aged than young men, especially under a high strain rate condition.

## Results

Table 1 shows the muscle thickness and twitch properties (measured using supramaximal electrical stimulation) in the two groups. No significant differences were observed in the muscle thickness of MG (p = 0.512, *d* = 0.217), LG (p = 0.836, *d* = 0.067), or SOL (p = 0.961, *d* = 0.015) between middle-aged and young men. Furthermore, there were no significant differences in peak twitch torque (p = 0.867, *d* = 0.055), time to peak twitch torque (p = 0.351, *d* = 0.309), or half relaxation time (p = 0.141, *d* = 0.493) between the two groups.

**Table 1:** Muscle thickness and twitch properties in middle-aged and young men Mean (sd).

	Middle-aged men	Young men
Mean muscle thickness of MG (mm)	21.7 (3.0)	21.0 (3.5)
Mean muscle thickness of LG (mm)	19.4 (2.6)	19.2 (3.3)
Mean muscle thickness of SOL (mm)	23.1 (4.7)	23.2 (3.8)
Peak twitch torque (Nm)	21.0 (3.9)	21.2 (4.4)
Time to peak twitch torque (ms)	119.7 (12.6)	115.8 (10.4)
Half relaxation time (ms)	100.7 (14.6)	93.4 (12.8)

**Figure 1:**
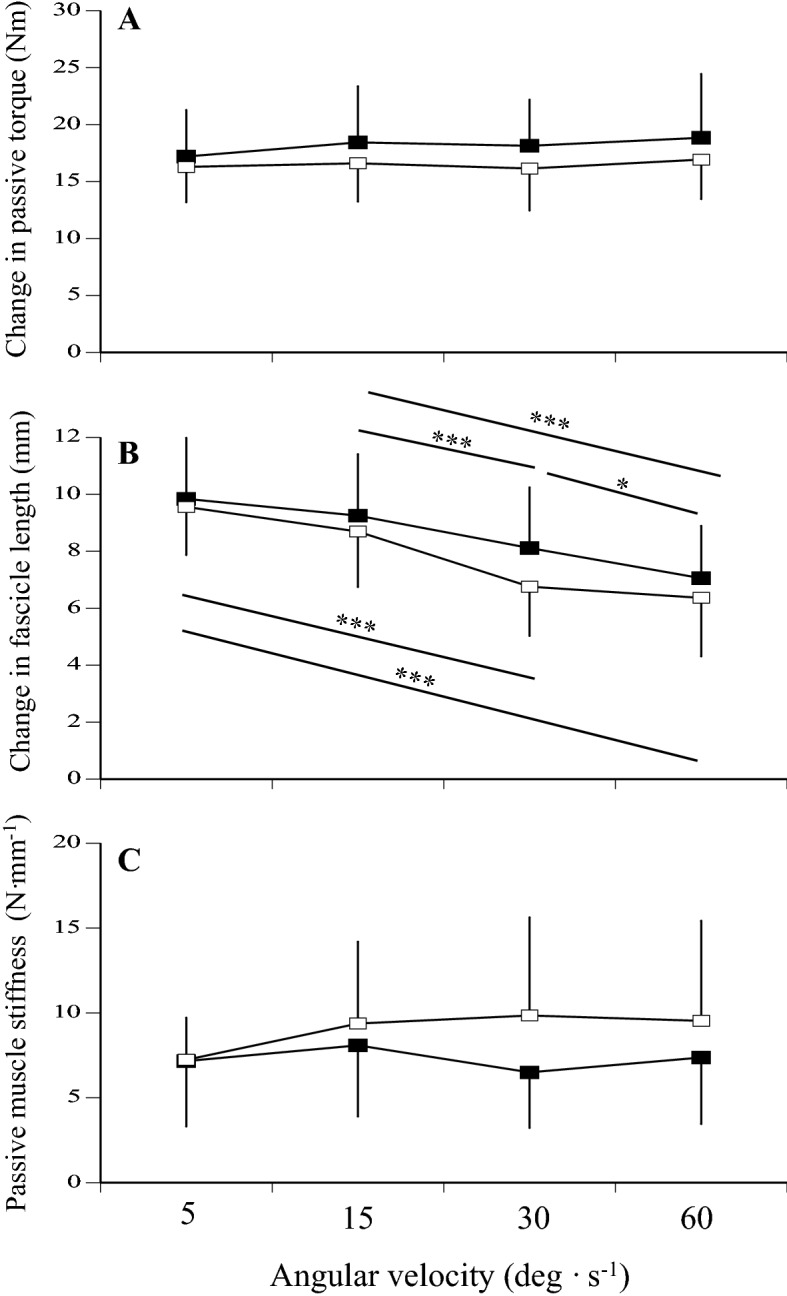
Changes in passive torque (**A**) and fascicle length (**B**), and passive muscle stiffness (**C**) at 5, 15, 30, and 60 deg s^−1^ in middle-aged (closed) and young (open) men. Significant difference among the conditions: *p < 0.05, ***p < 0.001.

The changes in passive torque, fascicle length, and passive muscle stiffness were measured during slow passive stretching at four different angular velocities (5, 15, 30, and 60 deg s^−1^). Regarding the change in passive torque during slow stretching, the effects of age (p = 0.180, pη^2^ = 0.057), angular velocity (p = 0.255, pη^2^ = 0.043), and interaction between age and angular velocity (p = 0.718, pη^2^ = 0.011) were not significant (Fig. 1A). Regarding the change in fascicle length during slow stretching, the effect of angular velocity (p < 0.001, pη^2^ = 0.617) was significant, although the effects of age (p = 0.233, pη^2^ = 0.045) and interaction between age and angular velocity (p = 0.257, pη^2^ = 0.043) were not (Fig. 1B). For both groups, the changes in fascicle length during slow stretching decreased as angular velocity increased. Concerning passive muscle stiffness, the effects of age (p = 0.130, pη^2^ = 0.069), angular velocity (p = 0.371, pη^2^ = 0.036), and interaction between age and angular velocity (p = 0.374, pη^2^ = 0.039) were not significant (Fig. 1C).

**Figure 2:**
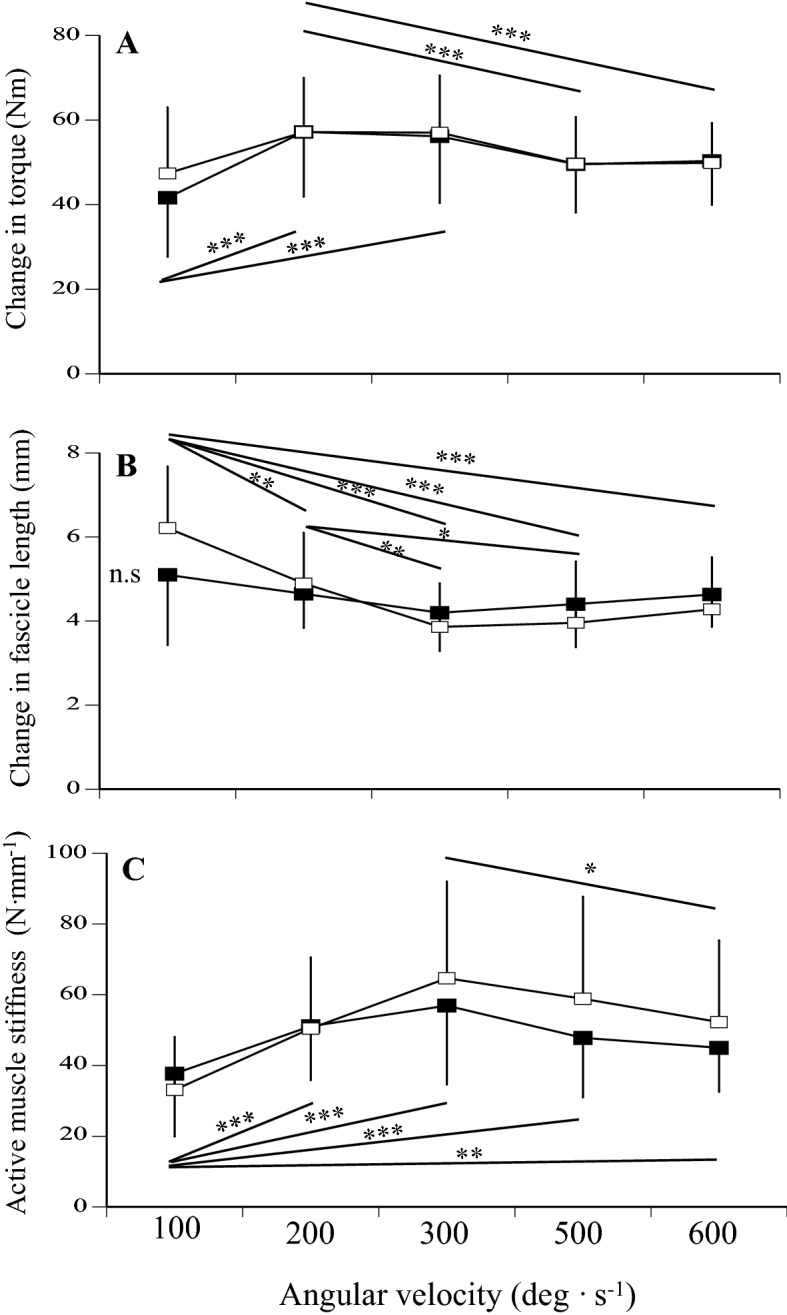
Changes in torque (**A**) and fascicle length (**B**), and active muscle stiffness (**C**) at 100, 200, 300, 500, and 600 deg s^−1^ in middle-aged (closed) and young (open) men. Significant difference among the conditions: *p < 0.05, **p < 0.01, ***p < 0.001.

The changes in torque, fascicle length, and active muscle stiffness were measured during fast stretching after submaximal isometric contraction at five different angular velocities (100, 200, 300, 500, and 600 deg s^−1^). Regarding the change in torque during fast stretching, the effect of angular velocity was significant (p < 0.001, pη^2^ = 0.450), whereas the effects of age (p = 0.760, pη^2^ = 0.003) and interaction between age and angular velocity (p = 0.190, pη^2^ = 0.046) were not (Fig. 2A). Regarding the change in fascicle length during fast stretching, the effects of angular velocity (p < 0.001, pη^2^ = 0.351) and interaction between age and angular velocity (p = 0.005, pη^2^ = 0.123) were significant, whereas the effect of age was not (p = 0.885, pη^2^ = 0.001) (Fig. 2B). No differences in the changes in fascicle length were noted among the five angular velocities for middle-aged men, although the changes in fascicle length decreased as the angular velocity increased from 100 to 300 deg s^−1^ in young men. Concerning active muscle stiffness, the effect of angular velocity was significant (p < 0.001, pη^2^ = 0.328), whereas the effects of age (p = 0.482, pη^2^ = 0.015) and interaction between age and angular velocity (p = 0.091, pη^2^ = 0.057) were not (Fig. 2C). For both groups, active muscle stiffness was greatest at 300 deg s^−1^ and decreased as the angular velocity increased or decreased.

**Figure 3:**
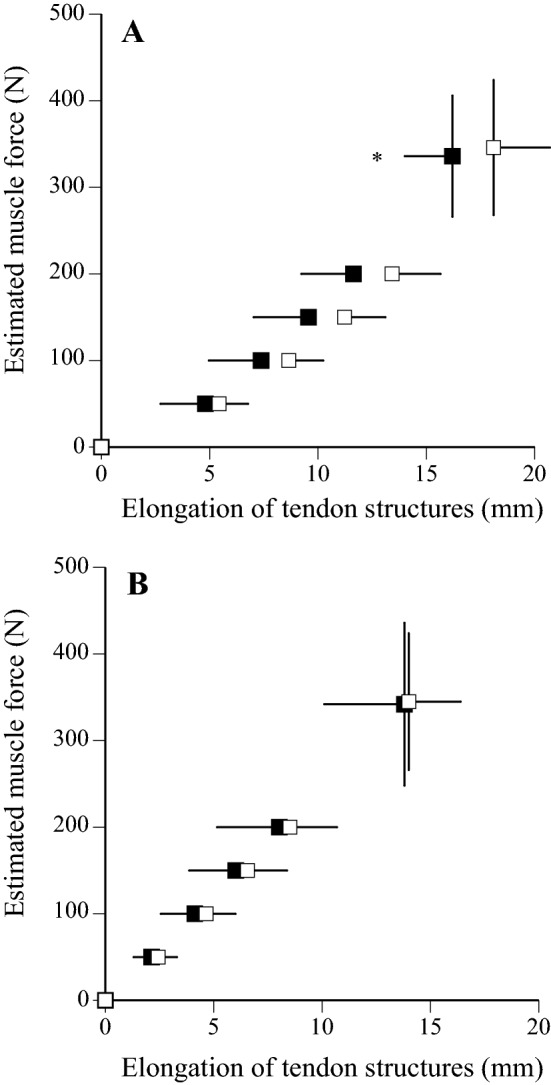
Relationship between estimated muscle force and elongation of tendon structures during ramp (**A**) and ballistic (**B**) contractions in middle-aged (closed) and young (open) men. Data are mean ± SD. Significant difference from young men: *p < 0.05.

**Figure 4:**
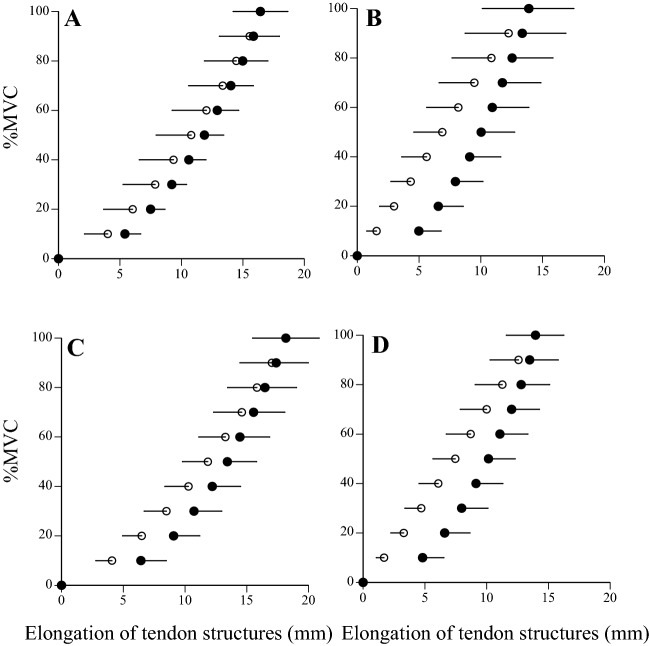
Relationship between %MVC and elongation of tendon structures during ramp (left: **A**,**C**) and ballistic (right: **B**,**D**) contractions in middle-aged (upper: **A**,**B**) and young (lower: **C**,**D**) men. Data are mean ± SD.

The mechanical properties of tendon structures were measured at two different strain rates (i.e., ramp and ballistic contractions). No significant differences were observed in maximal voluntary isometric contraction (MVC) during ramp (p = 0.636, *d* = 0.164) and ballistic (p = 0.844, *d* = 0.071) contractions between middle-aged (105 ± 22 Nm for ramp, 107 ± 30 Nm for ballistic) and young (109 ± 24 Nm for ramp, 109 ± 24 Nm for ballistic) men. Relationships between estimated muscle force and elongation of tendon structures during ramp and ballistic contractions are shown in Fig. 3. Maximal elongation of tendon structures during ramp contraction was significantly lower in middle-aged compared with young men (p = 0.032, *d* = 0.732), whereas no significant differences in elongation of tendon structures during ballistic contraction were noted between the two groups at any force production level (p = 0.830, *d* = 0.071) (Table 2). No significant differences in stiffness of tendon structures were found between the two groups (p = 0.660, *d* = 0.144 for ramp contraction, p = 0.801, *d* = 0.083 for ballistic contraction) (Table 2). Hysteresis of tendon structures during ballistic contraction tended to be greater in middle-aged than young men (p = 0.111, *d* = 0.534), although no difference in hysteresis of tendon structures during ramp contraction was noted between the two groups (p = 0.938, *d* = 0.025) (Fig. 4, Table 2). There was no difference in the tendon cross-sectional area between middle-aged (71.7 ± 13.2 mm^2^) and young (74.1 ± 11.9 mm^2^) men (p = 0.573, *d* = 0.185).

**Table 2:** Mechanical properties of tendon structures in middle-aged and young men Mean (sd).

	Middle-aged men	Young men
**Ramp**		
Maximal elongation (mm)	16.2 (2.2) *	18.1 (2.6)
Stiffness (N mm^−1^)	26.1 (9.1)	24.8 (8.0)
Hysteresis (%)	20.9 (14.5)	20.6 (10.8)
**Ballistic**		
Maximal elongation (mm)	13.8 (3.7)	14.0 (2.4)
Stiffness (N mm^−1^)	24.5 (10.5)	23.8 (5.1)
Hysteresis (%)	38.9 (13.3)	32.2 (10.9)

*Significantly different from young men (* p < 0.05).

Table 3 shows the variables measured during the two kinds of jumping tests using only the ankle joint (no-countermovement jump: noCMJ, countermovement jump: CMJ). For noCMJ and CMJ, no significant differences were observed in duration during eccentric (p = 0.613, *d* = 0.167 for CMJ) and concentric (p = 0.741, *d* = 0.109 for noCMJ; p = 0.497, *d* = 0.224 for CMJ) phases, angular velocities during eccentric (p = 0.330, *d* = 0.322 for CMJ) and concentric (p = 0.597, *d* = 0.174 for noCMJ; p = 0.639, *d* = 0.155 for CMJ) phases, mean power during concentric phase (p = 0.678, *d* = 0.087 for noCMJ; p = 0.863, *d* = 0.080 for CMJ), and jump height (p = 0.337, *d* = 0.318 for noCMJ; p = 0.952, *d* = 0.020 for CMJ) between middle-aged and young men.

**Table 3:** Measured variables during the two types of jumping tests Mean (sd).

	Middle-aged men	Young men
**noCMJ**		
Duration during concentric phase (ms)	278.4 (71.1)	270.8 (57.3)
Angular velocity during concentric phase (deg s^−1^)	159.7 (31.7)	165.3 (27.3)
Power during concentric phase (W kg^−1^)	3.4 (0.9)	3.2 (1.2)
Jump height (cm)	10.9 (1.2)	10.3 (2.0)
**CMJ**		
Duration during eccentric phase (ms)	425.9 (58.7)	434.8 (39.6)
Angular velocity during eccentric phase (deg s^−1^)	116.7 (22.0)	109.2 (21.1)
Duration during concentric phase (ms)	256.0 (47.3)	244.7 (45.7)
Angular velocity during concentric phase (deg s^−1^)	181.7 (30.1)	187.8 (40.9)
Power during concentric phase (W kg^−1^)	4.5 (0.9)	4.4 (1.4)
Jump height (cm)	13.9 (1.7)	13.9 (2.0)

*noCMJ* no-countermovement jump, *CMJ* countermovement jump.

## Discussion

The main results of the present study were that there were no differences in the passive and active muscle stiffness and tendon structures properties (except for maximal elongation of tendon structures during ramp contraction) measured at slow and fast strain rates for plantar flexors between middle-aged and young men. In addition, the measured variables during jumping using only the ankle joint for middle-aged men did not differ from those for young men.

For ramp contraction, maximal elongation of tendon structures of middle-aged men was significantly lower than that of young men. This result agreed with our previous finding^11^. However, we did not obtain the same result for ballistic contraction, contrary to our expectations. Our recent study showed that differences in elongation of tendon structures between ramp and ballistic contractions of the elderly were significantly smaller than those of young men^8^. In other words, this study suggested that tendon structures of the elderly would be lengthened excessively during rapid contractions. In the present study, however, differences in elongation of tendon structures between ramp and ballistic contractions of middle-aged men were similar to those of young men (data not shown). Therefore, we may say that age-related decline in power output for middle-aged men is not associated with age-related changes in extensibility of tendon structures, especially during rapid contractions, since there were no differences in elongation of tendon structures during ballistic contraction.

To date, few in vivo studies have attempted to investigate the effect of aging on hysteresis of tendon structures. Previous studies showed that hysteresis of tendon structures measured during ramp contraction increased with aging^8,10^. More recently, we demonstrated that hysteresis of tendon structures measured during ballistic contraction was considerably greater than that during ramp contraction^8,18^. Regarding the measurement of hysteresis of human tendons in vivo, Finni et al.^31^ pointed out that a very large range of hysteresis values for humans previously reported may be due to the difficulty in controlling the relaxation phase and a low sampling frequency of ultrasound images. Therefore, we need to consider a more reliable technique to measure hysteresis of the human tendons in vivo. In our previous studies, however, we obtained the similar results on hysteresis of tendon structures measured during ballistic contraction^18^. Furthermore, we found that no significant differences in hysteresis of tendon structures measured during ramp and ballistic contractions were noted between middle-aged and young men in the present study. An increase in hysteresis of tendon structures may theoretically lead to a decrease in reused elastic energy during stretch–shortening cycle exercises. Considering the present results on hysteresis of tendon structures, tendon viscocity of the middle-aged would not change as much as in the elderly^8^.

In the present study, there were no significant differences in passive muscle stiffness at any of the angular velocities between the two groups. This result was not consistent with previous findings on passive muscle stiffness of elderly subjects, which indicated greater passive torque for elderly people^20–23^. Previous researchers suggested that greater passive torque and stiffness of elderly people would be associated with increased collagen contents of the perimysium and endomysium^21,24^. Taking these previous findings into account together with the present results, it is likely that passive properties of middle-aged muscles have been changed less than those of the elderly reported previously.

Recently, we developed a specially designed motor-driven dynamometer that facilitated the attainment of higher angular velocities (i.e., 600 deg s^−1^). Using this dynamometer, we determined the effect of angular velocities on active muscle stiffness^29^. At the beginning of the present study, we expected active muscle stiffness at a high angular velocity to be lower for middle-aged than young men. However, there were no significant differences in active muscle stiffness at all angular velocities between middle-aged and young men (Fig. 2C). Although the reasons for the discrepancy are unknown, the exerted torque level during the measurement of active muscle stiffness (50% of MVC) may have affected the present results. Since the activation level during jumping was higher, we may need to determine active muscle stiffness at a higher torque level. Considering the present results on the muscle and tendon structures properties as mentioned above, we suggest that the sudden decline in power output for middle-aged people is not associated with the age-related changes in the mechanical properties of muscles and tendon structures. However, we will discuss this point later from another point of view.

No significant difference was noted in the measured twitch torque properties, especially time to peak torque, assessed by a supramaximal electrical stimulation between middle-aged and young men. Bosco and Komi^3^ stated that the very large decrease in jump performance could be due to the possible decrease in fast-twitch fibers with aging. Indeed, Larsson et al.^32^ reported that the relative distribution of type II fibers in a 40–49-year group was lower than that in a 20–29-year group. However, the present result on the muscle fiber composition (estimated from time to peak torque) did not agree with the previous finding. The reasons for this discrepancy may be related to differences in the measured sites, because previous studies on muscle fiber compositions investigated the vastus lateralis muscle^32^. At least, there may be no difference in muscle fiber compositions of the plantar flexor muscles between the two groups.

As described in “Introduction”, previous studies showed that the age-related decline of power output has already begun from middle age^3–6^. However, the present results on jump performances using only ankle joint were different from previous findings. The disagreement between the previous and present results may be due to differences in used joints during jumping. Although previous studies adopted a vertical jump (i.e., multi-joint exercise), subjects in this study performed jumps using an ankle-dominant strategy in order to accurately determine the effect of muscle and tendon structures properties on jump performance in middle-aged people. As a reason for this discrepancy, the effect of aging on the plantar flexor muscles may be small for middle-aged people. Indeed, previous studies demonstrated that the joint torque and the activation level of the plantar flexor muscles were higher than those of other lower limb muscles, e.g., hip extensors and knee extensors, during walking^33,34^. Therefore, previous results on age-related decline in vertical jump performances would be related to changes in muscle and tendon structures properties of hip and/or knee joints, but not plantar flexors, and the coordination among the joints. At present, we are trying to establish a method to measure active muscle stiffness in knee extensors in our laboratory. In a future study, we need to investigate the measured variables for knee extensors as with the present study for plantar flexors.

For the two decades, ultrasound-based methods have used to determine the mechanical properties of human muscles and tendons in vivo. However, some methodological considerations (e.g., incomplete scanning, tracking error, etc.) have been pointed out when these approaches are adopted to investigate behavior and responses of human muscles and tendons to various interventions^35^. In the present study, especially, we should notice that there are some limitations of methodology followed. Firstly, we used a previously reported moment arm length in order to calculate the muscle force. Unfortunately, information concerning the effect of aging on moment arm length has been limited^36^. Furthermore, it is difficult to obtain accurate moment arm length, because the moment arm length changes with increasing exerted muscle force and rotating joint^37^. We considered that there was no difference in the moment arm length between middle-aged and young men in the present study since there were no differences in the physical characteristics (height and lower leg length) between the two groups. Secondly, we defined the relative contribution of MG to force production as the percentage of physiological cross-sectional area of this muscle to that of plantar flexors^38^. In the present study, there were no differences in muscle thickness of each muscle between the two groups (Table 1). Therefore, we considered that the relative contribution of each constituent for torque production was similar between middle-aged and young men.

Unfortunately, the results of this study may not be generalized, since our subjects were only men. Some studies reported that the force-generating capacity of skeletal muscle depended on the menstrual cycle phase in women^39,40^. If we include women as the subjects, we need to measure the muscle and tendon properties and jump performance three times (menstrual, ovulatory, and luteal phases) per subject in order to reach a definite conclusion. In the future, it will be necessary to make measurements similar to this study for women (considering the influence of the menstrual cycle).

In conclusion, no significant differences in the passive and active muscle stiffness or tendon structures properties (except for maximal elongation of tendon structures during ramp contractions) measured for plantar flexors were noted between middle-aged and young men. In addition, no differences in jump performances using only the ankle joint were noted between the two groups, whereas the age-related decline in power output during the vertical jump (multi-joint exercises) was already observed in middle-aged men (e.g.^6^). In the future, therefore, we need to determine the mechanical properties of muscles and tendon structures in knee extensors of middle-aged people.

## Methods

### Subjects

The sample size was estimated using data from our previous study (Kubo et al. 2007a) in which the differences in maximal tendon elongation between middle-aged and young men were determined. On the basis of an α level of 0.05 and a power (1 − ß) of 0.8, it was shown that as least 17 subjects for each group were necessary for this study. Seventeen middle-aged (age: 40.8 ± 3.6 years, height: 173.9 ± 4.4 cm, body mass: 72.2 ± 9.0 kg, mean ± SD) and 21 young (age: 21.8 ± 1.9 years, height: 172.1 ± 4.4 cm, body mass: 70.0 ± 10.1 kg) men participated in this study. All subjects were sedentary or mildly active, with none involved in any type of exercise program. They were also free from cardiovascular and/or metabolic disorders according to a standardized interview. The procedures, purpose, and risks associated with the study were explained to all subjects, and they gave written informed consent to participate in this investigation before starting this project. This study was approved by the Local Ethics Committee of the Department of Sports Sciences, The University of Tokyo, and complied with their requirements for human experimentation. This study was conducted in accordance with the ethical principles stated in the Declaration of Helsinki.

### Muscle thickness and tendon cross-sectional area

An ultrasonic apparatus (SSD-900, Aloka, Japan) was used to determine the thickness of the medial gastrocnemius muscle (MG), lateral gastrocnemius muscle (LG), and soleus muscle (SOL) using a previously described procedure^41^. Cross-sectional images were obtained at proximal levels of 30% (MG and LG) and 50% (SOL) of the lower leg length. At that level, the mediolateral widths of MG and LG were determined over the skin surface, and the position of one-half of this width was used as the measurement site for each muscle. The position of greatest thickness in the lateral half of SOL was measured at the level described above. After the measurement of muscle thickness, the cross-sectional area of the Achilles tendon was also measured at the height of the lateral malleolus. The repeatability of measurements of muscle thickness and tendon cross-sectional area was confirmed in our previous study^41^.

### Passive muscle stiffness

Subjects lay prone on a test bench, and the waist and shoulders were secured by adjustable lap belts and held in position. The ankle joint was set at 100 deg (with the foot perpendicular to the tibia = 90 deg with angles more than 90 deg on plantar flexion) with the knee joint at full extension, and the foot tightly secured by two straps to the footplate of a specially designed dynamometer (T.K.K.S-18035, Takei Scientific Instruments Co., Ltd., Niigata, Japan). The passive torque and joint angle data were collected at a sampling rate of 1 kHz. Subjects did not warm up before the measurement of passive muscle stiffness. While subjects maintained completely relaxed muscles, the ankle was passively moved from 100 to 80 deg at four different angular velocities (angular velocities were 5, 15, 30, and 60 deg s^−1^). Unfortunately, we could not assess the passive muscle stiffness at higher angular velocities correctly since passive torque values changed suddenly at the start and end of motion due to the effect of inertia. In the present study, the absence of myoelectric activities of the LG, SOL, and tibialis anterior muscles using surface electromyography was confirmed. The order of tasks (5, 15, 30, and 60 deg s^−1^) was randomized to avoid any systematic effects. In order to minimize thixotropic effects as preconditioning^42,43^, we collected data after 5 cycles. The measurement of passive muscle stiffness was performed two times per condition (5, 15, 30, and 60 deg s^−1^). The measured values were the means of two tests. Passive torque (TQ) measured during slow stretching was converted to muscle force (Fm) using the following equation:

$$ {\text{Fm}} = {\text{k}} \cdot {\text{TQ}} \cdot {\text{MA}}^{{ - {1}}} $$

where k (0.16) represents the relative contribution of the physiological cross-sectional area of MG within the plantar flexor muscles^38^, and MA (50 mm) is the moment arm length of the triceps surae muscles at 90 deg of the ankle joint^37^.

A real-time ultrasonic apparatus (SSD-6500, Aloka, Tokyo, Japan) was used to obtain a longitudinal ultrasonic image of MG at the level of 30% of the lower leg length during slow stretching. Ultrasonic images were recorded on a videotape at 30 Hz and synchronized with recordings of a clock timer for subsequent analyses. The fascicle length was defined as the distance between the insertion of the fascicle into the superficial and deep aponeuroses.

The passive torque, joint angle, and fascicle length were measured during slow stretching. The slope of the portion of passive muscle force—fascicle length curve from 90 to 80 deg was defined as passive muscle stiffness^18,27^. The repeatability of measurement of passive muscle stiffness was confirmed in our previous study^26^.

### Active muscle stiffness

Subject’s posture and setup were the same for the measurement of passive muscle stiffness described above. After a standardized warm-up and submaximal contractions to get accustomed to the tests, subjects were asked to perform twice 3-s MVC for plantar flexion at a 100-deg ankle angle. The highest MVC value was used to determine the target torque during the measurement of active muscle stiffness.

After a 5-min rest period, subjects performed the measurements of active muscle stiffness at five different angular velocities (angular velocities were 100, 200, 300, 500, and 600 deg s^−1^) using a previously described procedure^29,30^. The dynamometer was programmed to apply dorsiflexion from 100 to 80 deg. The measurement of active muscle stiffness was performed three times per condition (100, 200, 300, 500, and 600 deg s^−1^) at 50% of MVC with the visual aid of exerted torque on an oscilloscope. If the pre-torque level was unstable, subjects were requested to perform additional measurements. During fast dorsiflexion, subjects were instructed to maintain the same perceived level of effort (under consciousness) during the dorsiflexion rotation. The order of tasks (100, 200, 300, 500, and 600 deg s^−1^) was randomized in order to avoid any systematic effects. Periods of 140, 80, 60, 48, and 48 ms after the onset of stretch were analyzed at 100, 200, 300, 500, and 600 deg s^−1^ in order to equalize the analyzed range of motion among five angular velocities (see Fig. 1 of Kubo et al.^29^). An additional measurement for each angular velocity was performed two times at 0% MVC. The averaged torque during the relaxed condition was subtracted from the measured torque during the active condition^26^. The measured values were the means of three tests.

**Figure 5:**
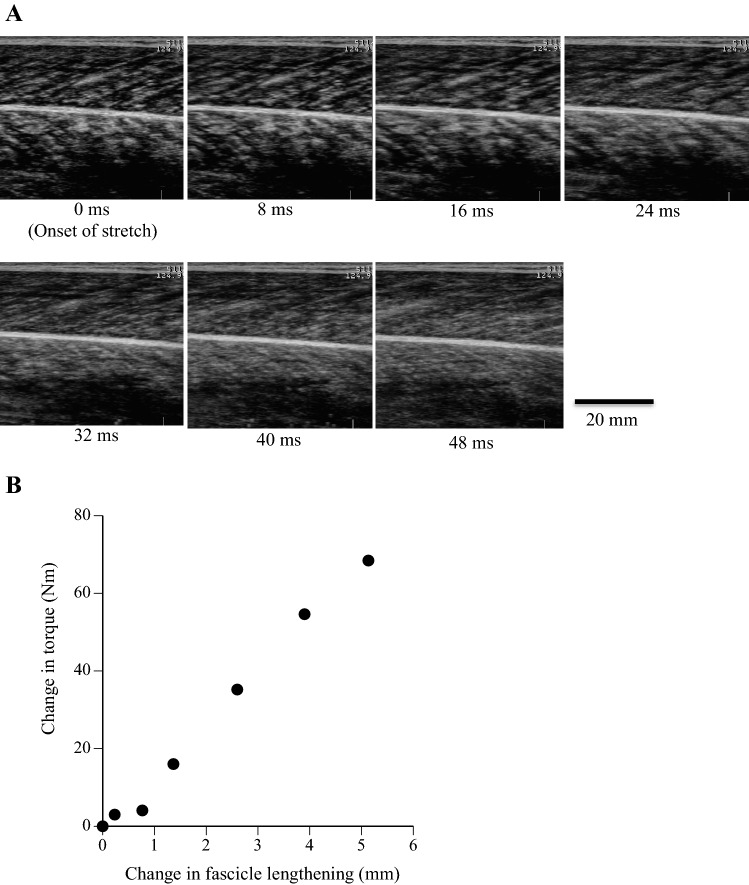
(**A**) Ultrasonic images of longitudinal sections of the medial gastrocnemius muscle during the measurement of active muscle stiffness at 600 deg s^−1^. (B) Typical example of the change in torque and fascicle lengthening during the measurement of active muscle stiffness at 600 deg s^−1^.

During the measurement of active muscle stiffness, fascicle length of MG was assessed using ultrasonic apparatus, as described in our previous studies^26,29,30^ (Fig. 5A). Ultrasonic images were stored at 100 Hz at 100, 200, and 300 deg s^−1^ and 125 Hz at 500 and 600 deg s^−1^ in the computer memory of the apparatus. The slope of muscle force–fascicle length was defined as active muscle stiffness^26^ (Fig. 5B). In our previous study with 8 young males^29^, the repeatability of measurement of active muscle stiffness was confirmed.

### Stiffness and hysteresis of tendon structures

Stiffness and hysteresis of tendon structures (outer-tendon and deep aponeurosis) during ramp and ballistic contractions were measured as described previously^18,30^. Subjects lay prone on the test bench of a dynamometer (custom made, VINE, Tokyo, Japan), with the foot tightly secured to the footplate of the dynamometer by two straps. The ankle joint was set at 90 deg with the knee joint at full extension. Prior to the test, subjects performed submaximal contractions to become accustomed to the test procedure. For ramp contraction, subjects were instructed to exert isometric torque from relaxation to MVC within 5 s, followed by gradual relaxation within 5 s. For ballistic contraction, subjects were instructed to exert isometric torque from relaxation to MVC powerfully and quickly, followed by sudden relaxation. However, the duration during ascending and descending phases (about 0.5 s) tended to be slightly longer than that in the previous studies (e.g.^44^), since subjects were requested to perform ballistic contractions to give priority to take clear ultrasonic images during measurements^8^. In addition, they asked to forbid moving the other parts except for plantar flexor muscles during the measurements. The measurement of tendon properties was performed twice per condition (ramp and ballistic contractions).

During the measurement of tendon structures properties, ultrasonic images were recorded on a videotape at 60 Hz and synchronized with recordings of a clock timer for subsequent analyses. Displacement of the point where one fascicle was attached to the middle part of aponeurosis was measured as elongation of tendon structures. However, displacement of the point where one fascicle was attached to the aponeurosis is also attributed to angular joint rotation occurred in the direction of ankle plantar flexion during isometric contraction^15^. An electrical goniometer (Penny and Giles, Newport, UK) was placed on the lateral aspect of the ankle in order to measure the ankle joint during measurements. To correct the measurements taken for elongation of tendon structures, additional measurements were performed under passive conditions from 0 to 9 deg. Displacement of the point where one fascicle was attached to the aponeurosis obtained from ultrasound images was corrected for that attributed to joint rotation alone^15^. Only values corrected for angular rotation are reported in the present study.

The torque measured by the dynamometer during isometric plantar flexion was converted to muscle force (Fm) described above. In the present study, the slope of muscle force and elongation of tendon structures above 50% of MVC was defined as stiffness of tendon structures^18^. The area within the force–elongation loop, as a percentage of the area beneath the curve during the ascending phase, was calculated as hysteresis of tendon structures^18^. The repeatability of measurements of tendon stiffness and hysteresis during ramp and ballistic contractions was confirmed in our previous study^18^.

### Twitch properties

The posture of subjects and procedure used were similar to those for the measurement of tendon structures properties described above. The torque data were collected at a sampling rate of 2 kHz. Twitch properties were measured using supramaximal electrical stimulation. The stimulating electrodes were placed on the skin of the popliteal fossa. A high-voltage stimulator (SEN-3301 with a specially modified isolator SS-1963, Nihon-Koden, Japan) generated rectangular pulses (single stimuli with a 500-µs duration for one stimulus). Maximal twitch contractions were evoked in the resting muscle by progressively increasing the stimulation intensity until increases failed to further elevate twitch torque. After a 5-min rest, peak twitch torque, time to peak twitch torque, and half relaxation time were measured twice, and the measured values reported below are the means of two trials. In our previous study with 8 young males^28^, the repeatability of measurement of twitch properties was confirmed.

### Jump performances

Subjects performed two types of unilateral maximal jumps using only the ankle joint (no-countermovement jump: noCMJ; countermovement jump: CMJ) on the sledge apparatus (AO-3000K, Applied Office, Japan). Due to scheduling limitations, only 32 of the 38 subjects (15 middle-aged and 17 young men) participated in the measurement of jump performances. The vertical component of the ground reaction force was recorded from a force plate (Kistler, 9281E, Switzerland) attached to the force platform of the apparatus at a sampling rate of 1 kHz. Retroreflective marks were placed on the fifth metatarsophalangeal joint, lateral malleolus, and lateral epicondyle of the knee. During jumping, subjects were filmed with a digital high-speed video camera at a sampling frequency of 250 Hz (VCC-H1600C, Digimo, Tokyo, Japan).

Subjects had adequate practices (around five times of submaximal jumps to become accustomed to the test procedures) of these two jumps before testing. In all tests, they were instructed to jump to a maximal height. The test was repeated five times per subject, with at least 2 min between trials. In noCMJ, subjects initially kept the ankle maximally dorsiflexed. Subjects then started ankle movement until the ankle was fully plantar-flexed and the toe lifted away from the force plate. In CMJ, subjects initially maintained the maximal plantar flexed position. Subjects then deactivated their plantar flexion torque to maximal dorsiflexion, and rebounded to start plantar flexion until the toe finally lifted away from the force plate.

The ankle joint angle and jump height were measured using open-source image analysis software (Image J, NIH, Bethesda, MD, USA). Jump height was defined as the maximum displacement of the seat of the sledge apparatus from the resting position (the ankle joint angle was 90 deg). The greatest jump height among five trials was used for the following analyses. The duration and mean angular velocities during eccentric (only CMJ) and concentric phases were calculated according to the ankle joint angle. In addition, ankle joint torque (TQ) during noCMJ and CMJ was calculated using the following equation^18,45^:

$$ {\text{TQ}} = {\text{Fz}} \cdot {\text{L1}} \cdot {\text{cos}}\;\left( {{\text{Aj}}} \right) $$

where Fz, L1, and Aj are the vertical component of the ground reaction force, the length from the estimated center of ankle joint to the ball of the foot (measured for each subject), and the ankle joint angle (see Fig. 3 of Kawami et al.^45^). Joint power (relative to body mass) was calculated by multiplying joint torque by joint angular velocity. In the present study, mean power during concentric phase was calculated according to the ankle joint angle.

### Statistical analysis

Descriptive data include means ± SD. Between-group analyses were conducted using an unpaired Student’s *t*-test. For an unpaired Student’s t-test, we confirmed for the normality of the data with the Kolmogorov–Smirnov test and checked for homogeneity of variance with Leven’s test. If the equal variance assumption was violated, we applied Welch’s t-test. A two-way analysis of variance (ANOVA) was used to detect significant effects of group and angular velocity on increments in torque, changes in fascicle length, and muscle stiffness. If the F statistic of the analysis of variance was significant, differences between means were assessed using Bonferroni’s post-hoc test. In ANOVA, Mauchly's sphericity test was performed to assess the homogeneity of variance. Greenhouse–Geisser correction was applied where the assumption of sphericity was violated. The effect size was calculated using partial eta-squared (pη^2^) for one- and two-way ANOVA and Cohen's *d* formula for an unpaired Student's t-test. The level of significance was set at p < 0.05.
